# A cross-sectional study of depression among women attending antenatal clinics in Blantyre district, Malawi

**DOI:** 10.4102/sajpsychiatry.v24i0.1181

**Published:** 2018-11-26

**Authors:** Genesis Chorwe-Sungani, Jennifer Chipps

**Affiliations:** 1 Kamuzu College of Nursing, University of Malawi, Malawi; 2 School of Nursing, University of the Western Cape, South Africa

## Abstract

**Background:**

Pregnancy is a period associated with major psychological and social changes in the life of a woman and can be associated with anxiety and depression.

**Aim:**

To describe demographic, clinical and risk profile of antenatal depression among pregnant women attending antenatal clinics in Blantyre district, Malawi.

**Setting:**

The study was conducted in eight antenatal clinics in Blantyre district, Malawi.

**Methods:**

A cross-sectional study of 480 randomly selected pregnant women attending antenatal clinics was conducted. Prevalence was determined using the Edinburgh Postnatal Depression Scale (EPDS) which was validated against a sub-sample using the Mini International Neuropsychiatric Interview. The risk factors of depression were assessed using the Pregnancy Risk Questionnaire. Data were analysed using descriptive statistics, Pearson chi-square test and binary logistic regression.

**Results:**

Prevalence of antenatal depression using the EPDS was 19% (95% CI 15.5% – 22.5%, *n* = 91) and was comparable to the Mini International Neuropsychiatric Interview (25.8% [95% CI = 17.5–34], *n* = 25). The key risk factors that predicted antenatal depression were: ‘being distressed by anxiety or depression for more than 2 weeks during this pregnancy’ (OR = 4.1 [2.1–7.9], *p* ≤ 0.001); ‘feeling that a relationship with partner is not an emotionally supportive one’ (OR = 3.5 [1.4–8.4], *p* = 0.01); ‘having major stresses, changes or losses in the course of this pregnancy’ (OR = 3.2 [1.7–6.2], *p* = 0.01); ‘feeling that father was critical of her when growing up’ (OR = 3.2 [1.4–7.6], *p* = 0.01); and ‘having history of feeling miserable or depressed for ≥2 weeks before this pregnancy’ (OR = 2.4 [1.3–4.4], *p* = 0.01).

**Conclusion:**

This study confirmed the high-prevalence rate of depression in this group and illustrated that antenatal depression was associated with being distressed by anxiety or depression; support from partner; major stresses during pregnancy; and history of feeling miserable or depressed before pregnancy. This study also found a history of poor relationship between pregnant women and their fathers during childhood.

## Introduction

Pregnancy is a period associated with major psychological and social changes in the life of a woman and can be associated with anxiety and depression.^1^ Depression may be a significant disease burden for pregnant women,^2^ is a leading cause of disability^3^ and can impact on functional status of a pregnant woman and the development of the foetus.^4^ Antenatal depression is also a risk factor for postnatal depression.^5^ Therefore, it is important to conduct holistic, comprehensive assessments of pregnant women that include identifying risk and protective factors for depression during this time.

The early detection of risk factors for antenatal depression may result in reduction of disease burden experienced by these women.^6^ Nonetheless, antenatal depression and associated risk and protective factors are often not assessed during pregnancy in low-resource settings^7^ because antenatal services usually focus more on physical health and do not routinely screen for mental health issues.^4^ Thus, data on the local prevalence of depression and its associated risk factors are needed to provide clinicians with clinically relevant, identifiable information to accurately assess pregnant women with depression.

Studies have shown that prevalence of antenatal depression ranges from as low as 4.8% in high-income countries^8^ to as high as 35.8% in sub-Saharan countries.^4^ Although the prevalence of antenatal depression and associated psychosocial risk factors have been extensively studied in other parts of the world, there remains a scarcity of similar studies in Malawi. Currently, there are no national epidemiological data for prevalence of depressive disorders among pregnant women in Malawi. Only one rural district study reported a prevalence of 21.1% major depression among pregnant women.^2^ This study aims to add to the data on depression in pregnant women in Malawi by investigating the prevalence of depression and its associated psychosocial risk factors among pregnant women attending antenatal clinics in Blantyre district, Malawi.

## Methods

### Design

A cross-sectional study was conducted with an aim to screen for depression and associated risk factors in pregnant women attending antenatal care services in eight public antenatal clinics (four urban and four rural in January to May 2016) in the Blantyre district, Malawi, using the Edinburgh Postnatal Depression Scale (EPDS) and the Pregnancy Risk Questionnaire (PRQ). In a sub-sample, depression diagnosis was determined using the Mini International Neuropsychiatric Interview (MINI) and the EPDS was validated.

### Sample

To determine the prevalence of depression in this setting, a sample size of 480 pregnant women was calculated using the formula: *N* = (TP + FN)/ (1-P),^9^ with *N* = 1593, sensitivity 96%, specificity 57%,^10^ confidence interval 95% and *p* = 21%.^2^ Inclusion criteria were: attending an antenatal service with a pregnancy of any stage, being 18 years old and above, agreeing voluntarily to participate in the study, giving a written consent before joining the study and being able to speak and understand Chichewa (a local language). Exclusion criteria were: having complications of pregnancy or a known mental disorder. To determine Diagnostic and Statistical Manual of Mental Disorders-IV (DSM-IV) criteria for major depressive disorder (MDD) and to validate the EPDS as a gold standard, a sub-sample of 100 pregnant women was calculated as adequate to validate the EPDS against the MINI with sample parameters of *p* = 21%,^2^ *N* = 480, error level 7% and confidence interval 95%.

### Materials

This study used a standardised depression screening instrument, namely the EPDS, and the validated PRQ to collect data on risk factors for pregnancy. Although a previous study in Malawi used the SRQ,^2^ this study was performed in a single clinic with a convenience sample. In this study, following a systematic review of accurate instruments for screening depression in low-resource settings,^11^ the EPDS was selected. The EPDS is a 10-item self-reporting questionnaire for screening postnatal depression^12^ which can also be used to screen for antenatal depression.^11^ The instrument has a maximum total score of 30 with a standard cut-off score of ≥ 10 for depression caseness.^13^ The EPDS targets depressive symptoms that an individual has experienced in the past 7 days.^12^ The MINI, a brief structured diagnostic interview for DSM-IV,^14^ was used as the ‘gold standard’ in generating psychiatric diagnoses using only the MDD module. The PRQ is an 18-item scale which is designed to assess psychosocial factors for depression during pregnancy. The instrument was designed to assess psychosocial risk factors for depression during pregnancy and used to predict antenatal or postnatal depression.^15^ The PRQ has a maximum total score of 90. Previously validated Chichewa language versions of the EPDS were used in this study.^5^ The PRQ and the MINI were translated into Chichewa by the researcher and a bilingual social worker through forward and backward translations.^16^

### Data collection

Research assistants trained in administration of the EPDS and PRQ collected the data from 480 pregnant women. They systematically picked every other third pregnant woman from the queue after randomly picking the first one. A sub-sample of 100 pregnant women drawn from the 480 respondents volunteered to undergo further interview using the MINI. A mental health nurse administered the MINI and was blind to the respondents’ initial screening outcomes.

### Data analysis

The International Business Machines (IBM) Statistical Package for Social Sciences (SPSS) version 22.0 was used to analyse data. Significance level for all tests was set at 95%. Descriptive statistics were used to summarise data. A diagnosis of MDD based on the MINI was assigned. An EPDS ‘case’ of depression, that is, being screened positive, was computed based on a cut-off score of ≥ 10. The EPDS cases were validated against the MDD diagnosis using standard sensitivity analysis. Differences in demographics and psychosocial risk factors between screen positives and negatives were compared using Pearson chi-square test. Associations between psychosocial risk factors and EPDS depression cases were tested using odds ratios (OR). Direct logistic regression was performed to assess the impact of demographic variables and psychosocial risk factors in screening positive for depression in pregnant women.

## Ethical consideration

The study received ethics approval from Senate Research and Ethics Committee at the University of the Western Cape and College of Medicine Research and Ethics Committee at University of Malawi. Institutional clearance was also granted by Blantyre District Health Office for the researcher to conduct this research in the district. Pregnant women who screened positive on the EPDS were referred to a psychiatric clinic.

## Results

### Demographics

A total of 480 out of a possible 496 respondents had the EPDS and PRQ administered (response rate of 96.8%). The age of respondents ranged from 18 to 43 years (mean 25.2 ± 5.5). The mean number of pregnancies per respondent was 2.4 ± 1.3 (range = 1–6 pregnancies), with a current mean gestation period of 26.7 weeks ± 7.4 (range = 5–40 weeks). More than half of the respondents were unemployed (52.5%, *n* = 252), had more than primary school education (53.8%, *n* = 256) and were from an urban area (65.2%, *n* = 313). Nearly all the respondents were supported by a partner (92.9%, *n* = 446). No significant differences were found between demographic data of the depression cases and non-depression cases except for marital status and occupation (approached significance) and no difference by clinical pregnancy variables (Table 1).

**TABLE 1:** Demographic and clinical characteristics of respondents (*n* = 480).

Item	EPDS						Statistic χ^2^	*p*	OR	95% CI	*p*
	Positive†			Negative‡							
	*n*	%	Mean s.d.	*n*	%	Mean s.d.
**Occupation**											
Unemployed	53	58.2	-	199	51.2	-	1.5	0.22	0.62	0.37–1	0.06*
Employed	38	41.8	-	90	48.8	-					
**Education level**											
Primary or none	46	50.5	-	176	45.2	-	0.84	0.3	0.79	0.48–1.3	0.36
Secondary or above	45	49.5	-	213	54.8	-					
**Marital status**											
Supported by partner	80	87.9	-	366	94.1	-	4.3	0.04*	3.2	1.4–7.6	0.01*
Not supported by partner	11	12.1	-	23	5.9	-					
**Setting**											
Urban	57	62.6	-	256	65.8	-	0.33	0.57	1.2	0.71–1.9	0.55
Rural	34	37.4	-	133	34.2	-					
Age in years	-	-	25.2 ± 4.9	-	-	25.1 ± 5.6	2.7	0.43	1.1	0.98–1.1	0.21
Gestation in weeks	-	-	27.7 ± 7.4	-	-	26.5 ± 7.4	6.4	0.09	1.0	0.99–1.1	0.19
Pregnancies	-	-	2.3 ± 1.2	-	-	2.4 ± 1.3	5.7	0.13	0.97	0.64–1.2	0.37

† , 19 %, *n* = 91;

‡ , 81%, *n* = 389.

Data = *n* (%) or mean ± standard deviation; OR, odds ratio; CI, confidence interval; EPDS, Edinburgh Postnatal Depression Scale.

* significance set at ≤ 0.05

### Sensitivity analysis

A total of 3 out of the 100 respondents in the sub-sample refused to be interviewed, resulting in a sample size of 97 (97%) – 48 screen positives and 49 screen negatives (mean = 27.7 ± 7.9 years). The prevalence of current MDD using the MINI was 25.8% (95.0% CI = 17.5% – 34.0%, *n* = 25), with no significant differences between demographic and clinical data of the MDD cases and non-MDD cases. The EPDS had a sensitivity of 68.0%, specificity of 88.0% and AUC = 0.85, using the MDD diagnosis as the gold standard (MINI), confirming its validity in measuring risk for antenatal depression.

### Antenatal depression and demographic risk factors

Using the EPDS, this study found rates for antenatal depression cases (screen positives) of 19.0% (95.0% CI 15.5% – 22.5%, *n* = 91) in pregnant women attending antenatal clinics in Blantyre district. A significantly higher proportion of EPDS cases reported that were not supported by a partner (12.1%, *n* = 366) compared to 5.9% (*n* = 80) non-cases (*p* = 0.01) (Table 1). The demographic characteristics of the EPDS cases were similar to the sub-sample MDD cases (urban areas [62.8% vs. 60.0%], secondary education or over [49.5% vs. 56.0%], not supported by a partner [12.1% vs. 12.0%]), except for unemployment which was higher in MDD cases (58.2% vs. 80.0%). Similarly, the mean number of pregnancies in the EPDS versus MDD cases were 2.3 versus 2.5 and mean gestations of 27.7 weeks for both.

### Psychosocial risk factors for antenatal depression

The PRQ was used to assess psychosocial risk factors associated with antenatal depression. There were significant differences between EPDS positive and negative screened cases in 12 of the psychosocial risk factors measured by PRQ (Table 2).

**TABLE 2:** Psychosocial risk factors associated with antenatal depression.

Pregnancy Risk Questionnaire item	EPDS				Statistic *χ*^2^	*p*	OR (95% CI)	*p*
	Positive†		Negative‡					
	*n*	%	*n*	%				
Feeling that mother was critical of her when growing up	84	92.3	353	90.7	0.22	0.64	052 (0.12–2.2)	0.37
Feeling that father was critical of her when growing up	78	85.7	295	75.8	4.2	0.04*	3.5 (0.97–12.8)	0.06*
Having trouble finishing jobs because of wanting to get it exactly right	59	64.8	189	48.6	7.8	0.01*	1.8 (0.92–3.4)	0.09
Being distressed by anxiety or depression for ≥ 2 weeks during this pregnancy	53	58.2	44	11.3	101	< 0.001*	4.2 (2.2–8.2)	< 0.001*
Having history of feeling miserable or depressed for ≥ 2 weeks before this pregnancy	45	49.5	85	21.9	28.5	< 0.001*	2.3 (1.2–4.3)	0.01*
Having major stresses, changes or losses in the course of this pregnancy	48	52.7	44	11.3	81.7	< 0.001*	3.5 (1.7–6.9)	< 0.001*
Was physically abused when growing up	34	37.4	64	16.5	19.8	< 0.001*	1.7 (0.82–3.4)	0.15
Feeling that will have no people to depend on for emotional support after giving birth	30	33.0	47	12.1	23.9	< 0.001*	1.9 (0.86–4.1)	0.12
Feeling that a relationship with partner is not an emotionally supportive one	25	27.5	20	5.1	43.3	< 0.001*	3.8 (1.5–9.5)	0.004*
Feeling that pregnancy has not been a positive experience	20	22.0	29	7.5	16.9	< 0.001*	1.6 (0.64–4.1)	0.3
Feeling that father was not emotionally supportive of her when growing up	15	16.5	83	21.3	1.1	0.3	0.98 (0.30–3.1)	0.98
Generally considers herself as a worrier	15	16.5	52	13.4	0.59	0.44	1 (0.40–2.5)	0.99
Feeling that mother is not emotionally supportive of her at present	11	12.1	46	11.8	0.01	0.94	0.45 (0.15–1.3)	0.15
Previously told by health professional that she was depressed or needed antidepressants	9	9.9	12	3.1	8.2	0.004*	2.3 (0.62–8.2)	0.22
Feeling that mother was not emotionally supportive of her when growing up	8	8.8	10	2.6	7.9	0.01*	3.9 (0.84–18.1)	0.08*
Not liking herself as a person	6	6.6	17	4.4	0.79	0.37	1.4 (0.40–4.9)	0.59
Was sexually abused when growing up	6	6.6	3	0.80	13.6	< 0.001*	4.1 (0.56–30.2)	0.17
Thinking that her mother was not happy to be a mother	4	44.4	8	2.1	1.7	0.19	0.27 (0.03–2.4)	0.24

EPDS, Edinburgh Postnatal Depression Scale; CI, confidence interval; OR, odds ratio.

† , 19%, *n* = 91;

‡ , 81%, *n* = 389.

* Significance set at ≤ 0.05

Edinburgh Postnatal Depression Scale screen positives were significantly associated (*p* < 0.05) with being ‘distressed by anxiety or depression for a period of 2 weeks or more during this pregnancy’ (χ^2^ = 101, *p* < 0.001) (OR = 4.2 [2.2–8.2], *p* < 0.001), experiencing ‘major stresses, changes or losses in the course of this pregnancy’ (χ^2^ = 81.7, *p* < 0.001) (OR = 3.5 [1.7–6.9], *p* < 0.001), were ‘physically abused when they were growing up’ (χ^2^ = 19.8, *p* < 0.001) (OR = 1.7 [82–3.4], *p* = 0.15), had a history of feeling ‘miserable or depressed for 2 weeks or more before this pregnancy’ (χ^2^ = 28.5, *p* < 0.001) (OR = 2.3 [1.2–4.3], *p* = 0.01), ‘feeling that father was critical of her when growing up’ (χ^2^ = 4.2, *p* = 0.04) (OR = 3.5 [0.97–12.8], *p* = 0.06), ‘having trouble finishing jobs because of wanting to get it exactly right’ (χ^2^ = 7.8, *p* = 0.01) (OR = 1.8 [0.92–3.4], *p* = 0.09), ‘feeling that will have no people to depend on for emotional support after giving birth’ (χ^2^ = 23.9, *p* < 0.001) (OR = 1.9 [0.86–4.1], *p* = 0.12), ‘feeling that a relationship with partner is not an emotionally supportive one’ (χ^2^ = 43.3, *p* < 0.001) (OR = 3.8 [1.5–9.5], *p* = 0.004), ‘feeling that pregnancy has not been a positive experience’ (χ^2^ = 16.9, *p* < 0.001) (OR = 1.6 [0.64–4.1], *p* = 0.3), ‘feeling that mother was not emotionally supportive of her when growing up’ (χ^2^ = 7.9, *p* = 0.01) (OR = 3.9 [0.84–18.1], *p* = 0.08), ‘previously told by health professional that she was depressed or needed antidepressants’ (χ^2^ =8.2, *p* = 0.004) (2.3 [0.62–8.2], *p* = 0.22) and ‘sexually abused when growing up’ (χ^2^ = 13.6, *p* < 0.001) (OR = 4.1 [0.56–30.2], *p* = 0.17) compared to screen negatives (Table 2).

### Multivariate analysis of EPDS score and other variables

A direct logistic regression model with 14 variables (2 demographic and 12 psychosocial risk factors with significant differences) was constructed. The model with the 14 variables was statistically significant (χ^2^ = 153.9, *p* < 0.001) and it correctly classified 87.7% of screen positives. Furthermore, the model showed that there were only five predictors of caseness for antenatal depression, namely: (1) ‘being distressed by anxiety or depression for more than 2 weeks during this pregnancy’ (OR = 4.1 [2.1–7.9], *p* £ 0.001); (2) ‘feeling that a relationship with partner is not an emotionally supportive one’ (OR = 3.5 [1.4–8.4], *p* = 0.01); (3) ‘having major stresses, changes or losses in the course of this pregnancy’ (OR = 3.2 [1.7–6.2], *p* = 0.01); (4) ‘feeling that father was critical of her when growing up’ (OR = 3.2 [1.4–7.6], *p* = 0.01); and (5) ‘having history of feeling miserable or depressed for ≥ 2 weeks before this pregnancy’ (OR = 2.4 [1.3–4.4], *p* = 0.01). This showed that respondents who had been distressed by anxiety or depression for 2 weeks or more during pregnancy had the highest likelihood (four times) of screening positive for antenatal depression in this study.

## Discussion

Depression is the third largest contributor to the global burden of disease in the world which is estimated at 4.3%.^3^ As such, the high burden of depression among pregnant women may hinder their effective utilisation of antenatal services^7^ and may result in poor birth outcomes. In addition, these women may face challenges in taking care of themselves mentally and physically^17^ and may have increased likelihood of developing postnatal depression.^6^

This is the second study to investigate depression and associated risk factors in antenatal care in Malawi. This study found a prevalence of 19.0% (95.0% CI 15.5% – 22.5%, *n* = 91) for antenatal depression in pregnant women attending eight antenatal clinics in Blantyre in Malawi. This is consistent with previous studies’ prevalence ranges reported for sub-Saharan Africa.^2,4^ A previous study by Stewart et al.^2^ in one district hospital clinic in Malawi reported prevalence of current major depressive episode and current major or minor depressive episode of 10.7% (95.0% CI 6.9% – 14.5%) and 21.1% (95.0% CI 15.5% – 26.6%), respectively, using the SRQ.^2^ The SRQ differs from EPDS because it consists of binary questions that are easily understood by illiterate individuals and it includes somatic symptoms.^5^ However, both the EPDS and SRQ were previously found to be valid instruments for measuring antenatal depression in Malawi although the EPDS was adapted to include use of visual prompts.^5^ Consistent with previous studies,^13^ our findings showed that the EPDS remains to be a valid instrument (sensitivity of 68.0%, specificity of 88.0% and AUC = 0.85) for detecting antenatal depression when used in its original form locally.

### Psychosocial risk factors associated with antenatal depression

This study found that depression is significantly associated with being alone, unemployment, major stresses, poor relationships, physical or sexual abuse, lack of support and prior history of anxiety or depression. There is evidence which shows that domestic violence, maternal anxiety, life stress, prior depression and lack of social support are psychosocial risk factors of antenatal depression.^6^ This study confirmed these factors. A personal history of depression and experiencing stress are risk factors that are associated with antenatal depression.^6^ The risk factor which was the strongest predictor of depression was ‘being distressed by anxiety or depression during pregnancy’, with respondents reporting this being four times more likely to screen positive for antenatal depression. Another risk factor that was associated with screening positive for antenatal depression was ‘having major stresses, changes or losses in the course of this pregnancy’ with respondents who had experienced major stresses, changes or losses during pregnancy being three times more likely to be depressed.

It is documented that antenatal depression is more likely to occur among pregnant women with a recent history of stressful life events.^17,18^ Major stresses such as the death of a relative or intimate partner violence may have contributed to their depressive symptoms. In addition, it has been postulated that childhood physical abuse is associated with depressive symptoms in early pregnancy.^19^ ‘Having history of physical abuse when growing up’ was significantly associated with antenatal depression in this study, although this was not a significant factor in the overall prediction model.

The thought of having an unsupportive partner was significant in this study, with ‘feeling that relationship with partner is not an emotionally supportive one’ being found to be a risk factor that predicted antenatal depression (OR = 3.5 [1.4–8.4], *p* = 0.01). This is supported by other studies that showed that a pregnant woman may suffer from depression if she lacks support from her partner.^20^ Most pregnant women attending antenatal clinics in low-resource settings depend on their partners for financial support^2^ and are at risk of depression if they do not receive adequate psychosocial support.^6^ Psychosocial support serves as a buffer from stressful life events by providing resources, support and strength during pregnancy.^21^

The relationship of children and their fathers during childhood may reduce or increase the children’s vulnerability to emotional problems. This study found that pregnant women who indicated that their fathers were critical of them when growing up were three times more likely to screen positive of antenatal depression. Consistent with this finding is Rosenberg and Wilcox who asserted that girls who had a good relationship with their fathers develop a stronger self-esteem and are less likely to experience depression.^22^ Therefore, this study suggests that poor relationship between father and girl child during childhood is a risk factor for antenatal depression.

### Study limitations

This study may have been affected by selection bias because pregnant women who did not present themselves at antenatal clinics were not represented. Secondly, the interviewer administration of screening instruments may have influenced respondents to give answers that they deemed as socially acceptable in the presence of the interviewer.

## Conclusion

This study showed that antenatal depression was associated with being distressed by anxiety or depression, lack of support from partner, major stresses during pregnancy and history of feeling miserable or depressed before pregnancy. A history of poor relationship between pregnant women and their fathers during childhood makes them vulnerable to antenatal depression in this population. Knowledge of risk factors for antenatal depression is important to enable midwives in low-resource settings to timely detect depression during pregnancy^5^ and implement relevant psychosocial interventions in order to reduce incidences of antenatal depression.
